# Wastewater Target Pathogens of Public Health Importance for Expanded Sampling, Houston, Texas, USA

**DOI:** 10.3201/eid3008.231564

**Published:** 2024-08

**Authors:** Komal Sheth, Loren Hopkins, Kaavya Domakonda, Lauren Stadler, Katherine B. Ensor, Catherine D. Johnson, Janeana White, David Persse, Edward Septimus

**Affiliations:** City of Houston Department of Health and Human Services, Houston, Texas, USA (K. Sheth, L. Hopkins, D. Persse, K. Domakonda, J. White);; Rice University, Houston (L. Hopkins, K. Ensor);; Rice University George R. Brown School of Engineering, Houston (L. Stadler);; Houston Health Foundation, Houston (C.D. Johnson);; Texas Children's Hospital, Houston (C.D. Johnson);; Memorial Hermann Health System, Houston (E. Septimus);; Harvard Medical School, Boston, Massachusetts, USA (E. Septimus)

**Keywords:** Wastewater surveillance, target pathogens, expanded sampling, wastewater-based epidemiological monitoring, public health, communicable diseases, pilot study, Houston, Texas, United States

## Abstract

Building on the success of initiatives put forth during the COVID-19 pandemic response, US health officials are expanding wastewater surveillance programs to track other target pathogens and diseases of public health interest. The Houston Health Department in Houston, Texas, USA, conducted a hypothesis-generating study whereby infectious disease subject matter experts suggested potential targets. This study addressed 2 criteria recommended by the National Academies of Sciences, Engineering, and Medicine for selecting wastewater targets. Results can be used as a basis of a questionnaire for a future population-based study to recommend targets of highest priority to include for expanded wastewater sampling.

During the COVID-19 pandemic, wastewater surveillance, the measurement of pathogen levels in wastewater, emerged as a critical tool used by public health authorities to monitor the SARS-CoV-2 virus (*1*). Wastewater data, when paired with traditional surveillance methods, are poised now to play an important role in informing disease prevention and mitigation strategies for additional target pathogens (*2*). In May 2020, the Houston Health Department (HHD; Houston, TX, USA), in collaboration with municipal and academic partners, established a comprehensive SARS-CoV-2 wastewater surveillance system for the city of Houston, which subsequently became a Centers for Disease Control and Prevention National Wastewater Surveillance System Center of Excellence (*3*).

## Purpose

The National Academies of Sciences, Engineering, and Medicine recommends selection of targets to expand wastewater surveillance beyond SARS-CoV-2 be based on 3 criteria: 1) public health significance, 2) analytical feasibility, and 3) usefulness of the wastewater surveillance data to inform public health action (*4*). The HHD, working with an existing advisory committee of local infectious disease doctors and public health specialists, conducted a hypothesis-generating study whereby infectious disease subject matter experts (SMEs) suggested a list of pathogens or diseases (hereafter referred to as targets) to be considered for surveillance with respect to criteria 1 and 3 above, considering feasibility for assessment outside the scope of the study (*5*).

## Methods

### Study Development and Implementation

The survey on which the study is based (Appendix 1) provides its intended purpose, indicates that de-identified results would be publicly shared in aggregate, and asks for consent to participate (Appendix 2). Upon consent, the participants provided contact information, academic credentials, and rankings of the 74 targets in terms of the public health importance and actionability for public health intervention (e.g., education, outreach, testing, vaccination). The target options were compiled from the HHD’s database of reportable diseases monitored in Houston, Texas (*6*,*7*). SARS-CoV-2 virus, required by the National Wastewater Surveillance System, was excluded from this list. Participants classified public health importance by most important, important, or less important, and actionability for public health intervention by actionable, somewhat actionable, not actionable, and don’t know. Finally, participants were asked to identify the top 3 most important targets from the list; they were also invited to provide comments and suggest additional targets not listed.

The study population was selected by using nonprobability methods, without use of quotas or incentives. Following recommendations from the advisory committee, the study team distributed invitations to participate in the study by using email listservs, newsletters, and online forums, including those affiliated with the Infectious Diseases Society of America, Big Cities Heath Coalition, and the Council of State and Territorial Epidemiologists, to encourage self-identified infectious disease SMEs to participate. The study period was February 28–August 31, 2023. Rice University’s Institutional Review Board reviewed and approved all study procedures. The study adheres to accepted public opinion research guidelines (*8*).

### Data Analysis

We exported survey responses from the Qualtrics platform (https://www.qualtrics.com) into Excel software. We checked survey responses for duplicates, using the participant’s name and organization, and completeness. We excluded responses with only the consent portion filled out but without any further completion of personal information or classification of targets.

We tallied response counts by category for each possible classification option and assigned numerical weights. In the Public Health Importance category, most important = 3 points, important = 2 points, and less important = 1 point. In the Actionable for Public Health Intervention category, actionable = 3 points, somewhat actionable = 2 points, not actionable option = 1 point, and don’t know = 0 points. We assigned don’t know responses 0 points to differentiate from nonresponses. We multiplied weights by the number of participant responses for each option and then totaled for each target in both categories. Category totals provided an overall total score for each target. We also calculated averages, total divided by the number of participant responses for each target (including don’t know responses), to better interpret results for targets when not all participants submitted responses for each of the 74 targets on the target list. We added the average scores for each category to obtain an overall average score for each target.

We sorted the overall total scores and overall average scores from highest to lowest to generate top 10 lists for suggested targets. We ranked targets 1–10 based on their score in each category (ranking tied scores equally) (Table). Because >1 target can have the same score, >10 suggested targets could be obtained.

**Table:** Wastewater targets as suggested by infectious disease subject matter experts who participated in a study of wastewater target pathogens of public health importance for expanded sampling, Houston, Texas, USA, February 28, 2023–August 31, 2023

Target	N*	Overall total score (ranking)†	Overall average score (ranking)‡
Influenza A, novel/variant	43	226 (1)	5.26 (2)
Measles (rubeola)	41	216 (2)	5.34 (1)
Hepatitis A	43	202 (3)	4.76 (5)
Carbapenem-resistant Enterobacterales	43	200 (4)	4.65 (6)
Monkeypox virus	41	194 (5)	4.79 (4)
*Neisseria meningitidis*, invasive (meningococcal disease)	38	188 (6)	4.95 (3)
*Candida auris*	42	185 (7)	4.40 (12)
West Nile virus	41	180 (8)	4.39 (13)
Rabies, human	39	180 (8)	4.62 (7)
Anthrax	39	179 (10)	4.59 (8)
Legionellosis	41	179 (10)	4.7 (15)
Pertussis	39	175 (14)	4.55 (9)
Cholera	39	173 (17)	4.50 (10)

*The number of infectious disease subject matter experts recruited using nonprobability methods, who provided a response in either of the Public Health Importance or Actionable for Public Health Intervention categories for this target. †Overall total score is the summed totals of the Public Health Importance and Actionable for Public Health Intervention categories, with the target’s ranking corresponding to this sum. ‡Overall average score is the summed totals of the averages (total score divided by the number of respondents for each target) for the Public Health Importance and Actionable for Public Health Intervention categories, with the target’s ranking corresponding to this sum.

## Results

The HHD received 47 unique and complete survey responses affiliated with 42 unique organizations (19 university or hospital systems, 21 public health departments, 2 others) from 21 different states, primarily in large cities or counties. Of the participants from public health departments, 19 worked at the city or county level and 2 worked at state-level agencies.

There was significant consistency across both categories. The suggested targets based on the 10 highest score values in either category were influenza A (novel or variant), measles (rubeola), hepatitis A, carbapenem-resistant Enterobacterales, monkeypox virus, *Neisseria meningitidis* (invasive [meningococcal disease]), *Candida auris*, West Nile virus, rabies (human), anthrax, legionellosis, pertussis, and cholera. Participants suggested several additional targets of concern to include in expanded wastewater monitoring, including norovirus, rotavirus, Marburg virus, and multidrug-resistant pathogens. Suggested target lists from academic, healthcare, and public health participants included measles, influenza A, hepatitis A, and *Neisseria meningitidis*, with an expected variance between lists relevant to each participant group’s healthcare focus.

## Discussion

The results of this study cannot be used to represent opinions regarding the prioritization of these suggested targets and cannot be generalized to a broader population, but they can be used to suggest a list of targets that could be considered for surveillance. As such, results from the study identified a list of 13 targets to be considered for expanded wastewater sampling based on public health importance and actionability. The results also can be used as the basis of a questionnaire for a future population-based study, with the intent of homing in on recommendations for target prioritization on a broader scale. Furthermore, supplementing these results with additional data based on local needs might favor the inclusion of different targets for a specific region.

A critical first step in expanding a wastewater surveillance program is to understand the pathogens that infectious disease SMEs consider to be the greatest threat to public health. A previous study ranked targets for wastewater surveillance prioritization by using binary and quantitative parameters based in empirical disease data (*9*). Other disease prioritization studies that incorporated feedback from SMEs were not focused specifically on rankings for wastewater surveillance and had narrower scopes focusing on specific events or types of targets (*10*,*11*). The methods used in this study complement those approaches by bringing in perspectives from infectious disease SMEs with a first-hand view of how these targets affect disease in the broader community, whose responses were provided with the express intent of translating the collective data to wastewater monitoring. We believe that these study results can be used to suggest an expanded target list for the National Wastewater Surveillance System and serve as pilot information for future studies.

Appendix 1 The Houston Wastewater Epidemiology group’s Wastewater Target Prioritization survey used for study of wastewater target pathogens of public health importance for expanded sampling, Houston, Texas, USA.

Appendix 2 Additional information for study of wastewater target pathogens of public health importance for expanded sampling, Houston, Texas, USA.
