# Supplementary material for: Wastewater Target Pathogens of Public Health Importance for Expanded Sampling, Houston, Texas, USA
Source: Emerg Infect Dis. 2024 Aug;30(8):e231564. doi: 10.3201/eid3008.231564 (PMC11286076; doi:10.3201/eid3008.231564)
Supplement: Appendix 1 — The Houston Wastewater Epidemiology group’s Wastewater Target Prioritization survey used for study of wastewater target pathogens of public health importance for expanded sampling, Houston, Texas, USA. [file 23-1564-Techapp-s1.pdf]

Article DOI: http://doi.org/10.3201/eid3008.231564

Wastewater Target Pathogens of Public Health Importance for Expanded Sampling, Houston, Texas, USA

Appendix 1

The Houston Wastewater Epidemiology group's Wastewater Target Prioritization survey used in this study is shown on the following pages.

HOUSTON HEALTH DEPARTMENT

Consent

The Houston Wastewater Epidemiology group, composed of the Houston Health Department, Rice University, and Houston Public Works, is looking to identify virus/pathogen targets to potentially include in wastewater sampling through a CDC-funded Wastewater Center of Excellence program. As part of this program, we are asking infectious disease subject matter experts (not wastewater laboratory analysis experts) to prioritize which infectious diseases to monitor in wastewater (e.g., the pathogens with the greatest public health importance and actionability to sample for in wastewater) using a brief survey. Please note that the purpose of this survey is not to determine whether these pathogens can be detected in wastewater via laboratory methods.

Once the survey results have been analyzed, the Houston Wastewater Epidemiology Group aims to disseminate the results to a wider audience, including project stakeholders, other jurisdictions that may be conducting wastewater sampling, and the general public. As such, we are asking your consent for your participation in the survey and to share the results of the survey.

Please note, your responses will remain de-identified and survey results will be shared in aggregate.

Participation in this survey is voluntary. If you have any questions, please do not hesitate to email the Houston Wastewater Epidemiology group at info@hou-wastewater-epi.org.

Note: This survey is included as part of IRB Protocol *IRB-FY2023-161.* The Principal

Investigator of this project is Dr. Loren Hopkins, PhD (Loren.Hopkins@houstontx.gov, 832-393-5155).

Do you consent to participate in the Houston Wastewater Epidemiology group's Wastewater Target Prioritization survey? Results from this survey will be made publicly available (e.g., publishing reports online to the Houston Wastewater Epidemiology group's website, distributing via email, etc.).

- ◯ Yes
- ◯ No

Please enter your name and today's date to complete the consent.

Name of Participant	
Date (MM/DD/YYYY)	

Consent - Yes

The viruses and diseases included in this survey come from the Houston Electronic Disease Surveillance System, which is used to track **reportable** diseases in Houston.

We invite you to complete this brief survey in which you will identify wastewater targets to prioritize, evaluate their public health importance and if they are actionable for public health intervention, and determine at which level(s) these targets should be sampled. The answers from this survey will help to inform program actions. Thank you.

Please enter your name, your organization affiliation, location, and your email address.

Name

Affiliation

City, State

Email address

Please enter your credentials and/or specialty, as best fits your field of expertise (e.g., MD - Infectious Disease; MD - Internal Medicine; PhD - Infectious Disease; etc). Please select all that apply.

- [ ] MD/DO
- [ ] PhD
- [ ] MPH
- [ ] DrPH
- [ ] Other (Please describe)

For each of the viruses/pathogens, please rank their importance in terms of 1) public health importance, and 2) actionable for public health intervention (e.g., education/outreach, testing, vaccination).

	Public Health Importance			Actionable for Public Health Intervention			
	Most Important	Important	Less Important	Actionable	Somewhat Actionable	Not Actionable	Don't Know
Acute Flaccid Myelitis (AFM)	○	○	○	○	○	○	○
Amebiasis	○	○	○	○	○	○	○
Amebic Meningoencephalitis, Primary (PAM)	○	○	○	○	○	○	○
Ancylostomiasis (Hookworm)	○	○	○	○	○	○	○
Anthrax	○	○	○	○	○	○	○
Ascariasis	○	○	○	○	○	○	○
Aseptic (viral) Meningitis	○	○	○	○	○	○	○
Babesiosis	○	○	○	○	○	○	○
Bacterial and Other Meningitis	○	○	○	○	○	○	○
Botulism, Infant	○	○	○	○	○	○	○
Brucellosis	○	○	○	○	○	○	○
California Serogroup Virus, Neuroinvasive	○	○	○	○	○	○	○
Campylobacteriosis	○	○	○	○	○	○	○
Candida Auris	○	○	○	○	○	○	○
Carbapenem-resistant Enterobacteriaceae (CRE)	○	○	○	○	○	○	○
Chagas	○	○	○	○	○	○	○
Chikungunya Virus Disease	○	○	○	○	○	○	○
Cholera	○	○	○	○	○	○	○
Creutzfeldt-Jakob Disease	○	○	○	○	○	○	○
Cryptosporidiosis	○	○	○	○	○	○	○
Cyclosporiasis	○	○	○	○	○	○	○
Cysticercosis	○	○	○	○	○	○	○
Dengue	○	○	○	○	○	○	○

	Public Health Importance			Actionable for Public Health Intervention			
	Most Important	Important	Less Important	Actionable	Somewhat Actionable	Not Actionable	Don't Know
Ehrlichiosis, Chaffeensis	◯	◯	◯	◯	◯	◯	◯
Encephalitis, Nonarboviral	◯	◯	◯	◯	◯	◯	◯
Flavivirus	◯	◯	◯	◯	◯	◯	◯
Haemophilus Influenzae, Invasive	◯	◯	◯	◯	◯	◯	◯
Hantavirus Pulmonary Syndrome (HPS)	◯	◯	◯	◯	◯	◯	◯
Hemolytic Uremic Syndrome	◯	◯	◯	◯	◯	◯	◯
Hepatitis A	◯	◯	◯	◯	◯	◯	◯
Hepatitis B	◯	◯	◯	◯	◯	◯	◯
Hepatitis C	◯	◯	◯	◯	◯	◯	◯
Hepatitis E	◯	◯	◯	◯	◯	◯	◯
Influenza A, Novel/Variant	◯	◯	◯	◯	◯	◯	◯
Legionellosis	◯	◯	◯	◯	◯	◯	◯
Leishmaniasis	◯	◯	◯	◯	◯	◯	◯
Listeriosis	◯	◯	◯	◯	◯	◯	◯
Lyme Disease	◯	◯	◯	◯	◯	◯	◯
Malaria	◯	◯	◯	◯	◯	◯	◯
Measles (Rubeola)	◯	◯	◯	◯	◯	◯	◯
Methicillin-Resistant Staphylococcus Aureus (MRSA)	◯	◯	◯	◯	◯	◯	◯
Monkeypox	◯	◯	◯	◯	◯	◯	◯
Multidrug-Resistant Acinetobacter (MDR-A)	◯	◯	◯	◯	◯	◯	◯
Multisystem Inflammatory Syndrome-Children (MIS-C)	◯	◯	◯	◯	◯	◯	◯
Mumps	◯	◯	◯	◯	◯	◯	◯

	Public Health Importance			Actionable for Public Health Intervention			
	Most Important	Important	Less Important	Actionable	Somewhat Actionable	Not Actionable	Don't Know
Neisseria Meningitidis, Invasive (Meningococcal Disease)	○	○	○	○	○	○	○
Pertussis	○	○	○	○	○	○	○
Q Fever	○	○	○	○	○	○	○
Rabies, Human	○	○	○	○	○	○	○
Rickettsia, Unspecified	○	○	○	○	○	○	○
Rubella	○	○	○	○	○	○	○
Salmonella	○	○	○	○	○	○	○
Shiga Toxin-producing Escherichia coli (STEC)	○	○	○	○	○	○	○
Shigellosis	○	○	○	○	○	○	○
Spotted Fever Rickettsiosis	○	○	○	○	○	○	○
St. Louis Encephalitis, Non-Neuroinvasive	○	○	○	○	○	○	○
Streptococcal Toxic-Shock Syndrome	○	○	○	○	○	○	○
Streptococcus Pneumoniae, Invasive Disease (IPD)	○	○	○	○	○	○	○
Streptococcus, Invasive, Group A	○	○	○	○	○	○	○
Streptococcus, Invasive, Group B	○	○	○	○	○	○	○
Streptococcus, Invasive, Other	○	○	○	○	○	○	○
Taeniasis	○	○	○	○	○	○	○
Trichinosis (Trichinellosis)	○	○	○	○	○	○	○
Trichuriasis	○	○	○	○	○	○	○
Typhoid Fever (Salmonella Typhi)	○	○	○	○	○	○	○

	Public Health Importance			Actionable for Public Health Intervention			
	Most Important	Important	Less Important	Actionable	Somewhat Actionable	Not Actionable	Don't Know
Typhus Fever (Fleaborne)	◯	◯	◯	◯	◯	◯	◯
Vancomycin-Intermediate Staph Aureus (VISA)	◯	◯	◯	◯	◯	◯	◯
Varicella (Chickenpox)	◯	◯	◯	◯	◯	◯	◯
Vibrio Parahaemolyticus	◯	◯	◯	◯	◯	◯	◯
Vibrio Vulnificus Infection	◯	◯	◯	◯	◯	◯	◯
Vibriosis, Other/Unspecified	◯	◯	◯	◯	◯	◯	◯
West Nile Virus	◯	◯	◯	◯	◯	◯	◯
Yersiniosis	◯	◯	◯	◯	◯	◯	◯
Zika Virus	◯	◯	◯	◯	◯	◯	◯

Next, we are interested to see if there are any wastewater targets that we should be considering specifically for children (e.g., sampling at schools) and for congregate living facilities (e.g., nursing homes, detention centers, etc.), or if this target should be sampled city-wide. Please select at which specific level(s) the wastewater sampling should be conducted for each of the potential targets.

	Schools (K-12)	Nursing Homes	Jails/Detention Centers	Homeless Shelters	City-Wide
Acute Flaccid Myelitis (AFM)	☐	☐	☐	☐	☐
Amebiasis	☐	☐	☐	☐	☐
Amebic Meningoencephalitis, Primary (PAM)	☐	☐	☐	☐	☐
Ancylostomiasis (Hookworm)	☐	☐	☐	☐	☐
Anthrax	☐	☐	☐	☐	☐
Ascariasis	☐	☐	☐	☐	☐
Aseptic (viral) Meningitis	☐	☐	☐	☐	☐
Babesiosis	☐	☐	☐	☐	☐

	Schools (K-12)	Nursing Homes	Jails/Detention Centers	Homeless Shelters	City-Wide
Bacterial and Other Meningitis	☐	☐	☐	☐	☐
Botulism, Infant	☐	☐	☐	☐	☐
Brucellosis	☐	☐	☐	☐	☐
California Serogroup Virus, Neuroinvasive	☐	☐	☐	☐	☐
Campylobacteriosis	☐	☐	☐	☐	☐
Candida Auris	☐	☐	☐	☐	☐
Carbapenem-resistant Enterobacteriaceae (CRE)	☐	☐	☐	☐	☐
Chagas	☐	☐	☐	☐	☐
Chikungunya Virus Disease	☐	☐	☐	☐	☐
Cholera	☐	☐	☐	☐	☐
Creutzfeldt-Jakob Disease	☐	☐	☐	☐	☐
Cryptosporidiosis	☐	☐	☐	☐	☐
Cyclosporiasis	☐	☐	☐	☐	☐
Cysticercosis	☐	☐	☐	☐	☐
Dengue	☐	☐	☐	☐	☐
Ehrlichiosis, Chaffeensis	☐	☐	☐	☐	☐
Encephalitis, Nonarboviral	☐	☐	☐	☐	☐
Flavivirus	☐	☐	☐	☐	☐
Haemophilus Influenzae, Invasive	☐	☐	☐	☐	☐
Hantavirus Pulmonary Syndrome (HPS)	☐	☐	☐	☐	☐
Hemolytic Uremic Syndrome	☐	☐	☐	☐	☐
Hepatitis A	☐	☐	☐	☐	☐
Hepatitis B	☐	☐	☐	☐	☐
Hepatitis C	☐	☐	☐	☐	☐
Hepatitis E	☐	☐	☐	☐	☐
Influenza A, Novel/Variant	☐	☐	☐	☐	☐
Legionellosis	☐	☐	☐	☐	☐
Leishmaniasis	☐	☐	☐	☐	☐

	Schools (K-12)	Nursing Homes	Jails/Detention Centers	Homeless Shelters	City-Wide
Listeriosis	☐	☐	☐	☐	☐
Lyme Disease	☐	☐	☐	☐	☐
Malaria	☐	☐	☐	☐	☐
Measles (Rubeola)	☐	☐	☐	☐	☐
Methicillin-Resistant Staphylococcus Aureus (MRSA)	☐	☐	☐	☐	☐
Monkeypox	☐	☐	☐	☐	☐
Multidrug-Resistant Acinetobacter (MDR-A)	☐	☐	☐	☐	☐
Multisystem Inflammatory Syndrome-Children (MIS-C)	☐	☐	☐	☐	☐
Mumps	☐	☐	☐	☐	☐
Neisseria Meningitidis, Invasive (Meningococcal Disease	☐	☐	☐	☐	☐
Pertussis	☐	☐	☐	☐	☐
Q Fever	☐	☐	☐	☐	☐
Rabies, Human	☐	☐	☐	☐	☐
Rickettsia, Unspecified	☐	☐	☐	☐	☐
Rubella	☐	☐	☐	☐	☐
Salmonella	☐	☐	☐	☐	☐
Shiga Toxin-producing Escherichia coli (STEC)	☐	☐	☐	☐	☐
Shigellosis	☐	☐	☐	☐	☐
Spotted Fever Rickettsiosis	☐	☐	☐	☐	☐
St. Louis Encephalitis, Non-Neuroinvasive	☐	☐	☐	☐	☐
Streptococcal Toxic-Shock Syndrome	☐	☐	☐	☐	☐
Streptococcus Pneumoniae, Invasive Disease (IPD)	☐	☐	☐	☐	☐
Streptococcus, Invasive, Group A	☐	☐	☐	☐	☐
Streptococcus, Invasive, Group B	☐	☐	☐	☐	☐
Streptococcus, Invasive, Other	☐	☐	☐	☐	☐
Taeniasis	☐	☐	☐	☐	☐

	Schools (K-12)	Nursing Homes	Jails/Detention Centers	Homeless Shelters	City Wide
Trichinosis (Trichinellosis)	☐	☐	☐	☐	☐
Trichuriasis	☐	☐	☐	☐	☐
Typhoid Fever (Salmonella Typhi)	☐	☐	☐	☐	☐
Typhus Fever (Fleaborne)	☐	☐	☐	☐	☐
Vancomycin-Intermediate Staph Aureus (VISA)	☐	☐	☐	☐	☐
Varicella (Chickenpox	☐	☐	☐	☐	☐
Vibrio Parahaemolyticus	☐	☐	☐	☐	☐
Vibrio Vulnificus Infection	☐	☐	☐	☐	☐
Vibriosis, Other/Unspecified	☐	☐	☐	☐	☐
West Nile Virus	☐	☐	☐	☐	☐
Yersiniosis	☐	☐	☐	☐	☐
Zika Virus	☐	☐	☐	☐	☐

Please identify what you consider to be the Top 3 most important viruses/pathogens that we should sample for.

Virus/Pathogen 1 ________

Virus/Pathogen 2 ________

Virus/Pathogen 3 ________

If you have any comments about the rankings for the targets in this survey, please include them here.

If there are any additional wastewater virus/pathogen targets that have emerged since COVID-19 (e.g., 2020 onwards) that you would like us to consider for sampling, please list them below and at which level (e.g., city-wide, school, nursing home, etc.).

Consent - No

Thank you for your response. If you change your mind and wish to participate in the survey, you can click the "Restart Survey" button.

If you have any questions, please do not hesitate to email the Houston Wastewater Epidemiology group at info@hou-wastewater-epi.org.

- ◯ Restart Survey
- ◯ Exit Survey

Created by the Houston Health Department

Powered by Qualtrics
