# Supplementary material for: Wastewater Target Pathogens of Public Health Importance for Expanded Sampling, Houston, Texas, USA
Source: Emerg Infect Dis. 2024 Aug;30(8):e231564. doi: 10.3201/eid3008.231564 (PMC11286076; doi:10.3201/eid3008.231564)
Supplement: Appendix 2 — Additional information for study of wastewater target pathogens of public health importance for expanded sampling, Houston, Texas, USA. [file 23-1564-Techapp-s2.pdf]

Article DOI: http://doi.org/10.3201/eid3008.231564

Wastewater Target Pathogens of Public Health Importance for Expanded Sampling, Houston, Texas, USA

Appendix 2

Study Invitation Script

The authors drafted a script inviting infectious disease subject matter experts to participate in the study for distribution via the listservs, newsletters, etc., with the purpose of describing the study's purpose as well as emphasizing the intent to include perspectives from infectious disease subject matter experts. The script that was distributed is as follows:

"Wastewater surveillance has become a critical tool in monitoring the spread and burden of various diseases in the community, as evidenced by its success during the COVID-19 pandemic response to help direct public health action and interventions. The Houston Wastewater Epidemiology group is trying to identify pathogens of epidemiologic importance to public health to potentially include in expanded wastewater sampling through a CDC-funded Wastewater Center of Excellence program. As part of this program, we are asking infectious disease subject matter experts (not wastewater laboratory analysis experts) to prioritize which infectious diseases to monitor in wastewater (e.g., the pathogens with the greatest public health importance and actionability to sample for in wastewater) using a brief survey. Please note that the purpose of this survey is not to determine whether these pathogens can be detected in wastewater via laboratory methods.

Once the survey results have been collected and analyzed, the Houston Wastewater Epidemiology Group plans to disseminate the results to a wider audience. The results from this survey will guide other jurisdictions in determining which wastewater targets are of the highest public health importance to sample for, and shape wastewater monitoring nationally.

If you are interested in participating, please use the survey link below to submit a response by August 31, 2023, and feel free to share the survey link with your network. If you have any questions, please do not hesitate to email the Houston Wastewater Epidemiology group at info@hou-wastewater-epi.org. Thank you.

Survey link: https://houstonhealth.co1.qualtrics.com/jfe/form/SV_02hfVMXO8LQ9ise"

Target List

The Houston Health Department (HHD) included 74 target pathogens and diseases in the study, as identified from the HHD's database of reportable diseases that are monitored in Houston. This target list comprises the following. The names are written exactly as they were presented in the study, and they are presented in the same order as in the study.

Acute Flaccid Myelitis (AFM)

Amebiasis

Amebic Meningoencephalitis, Primary (PAM)

Ancylostomiasis

Anthrax

Ascariasis

Aseptic (viral) Meningitis

Babesiosis

Bacterial and Other Meningitis

Botulism, Infant

Brucellosis

California Serogroup Virus, Neuroinvasive

Campylobacteriosis

Candida Auris

Carbapenem-resistant Enterobacteriaceae (CRE)

Chagas

Chikungunya Virus Disease

Cholera

Creutzfeldt-Jakob Disease

Cryptosporidiosis

Cyclosporiasis

Cysticercosis

Dengue

Ehrlichiosis Chaffeensis

Encephalitis, Nonarboviral

Flavivirus

Haemophilius Influenzae, Invasive

Hantavirus Pulmonary Syndrome (HPS)

Hemolytic Uremic Syndrome

Hepatitis A

Hepatitis B

Hepatitis C

Hepatitis E

Influenza A, Novel/Variant

Legionellosis

Leishmaniasis

Listeriosis

Lyme Disease

Malaria

Measles (Rubeola)

Methicillin-Resistant Staphylococcus Aureus (MRSA)

Monkeypox

Multidrug-Resistant Acinetobacter (MDR-A)

Multisystem Inflammatory Syndrome-Children (MIS-C)

Mumps

Neisseria Meningitidis, Invasive (Meningococcal Disease)

Pertussis

Q Fever

Rabies, Human

Rickettsia, Unspecified

Rubella

Salmonella

Shiga Toxin-producing Escheria Coli (STEC)

Shigellosis

Spotted Fever Rickettsia

St. Louis Encephalitis, Non-Neuroinvasive

Streptococcal Toxic-Shock Syndrome

Streptococcus Pneumoniae, Invasive Disease (IPD)

Streptococcus, Invasive, Group A

Streptococcus, Invasive, Group B

Streptococcus, Invasive, Other

Taeniasis

Trichinosis (Trichinellosis)

Trichuriasis

Typhoid Fever (Salmonella Typhi)

Typhus Fever (Fleaborne)

Vancomycin-Intermediate Staph Aureus (VISA)

Varicella (Chickenpox)

Vibrio Parahaemolyticus

Vibrio Vulnificus Infection

Vibriosis, Other/Unspecified

West Nile Virus

Yersiniosis

Zika Virus

Survey

The Houston Wastewater Epidemiology group's Wastewater Target Prioritization survey contained 7 questions (Appendix 2). An introductory paragraph included a description of the study's purpose, as well as a statement that results would be made publicly available (with responses de-identified and shared in the aggregate). Invited participants were required to indicate their consent to participate. Those who declined to participate were given the option to change their response and proceed with the study or to confirm their refusal. Invited participant who confirmed their refusal were directed to the study's exit message.

The first question collected respondents' contact information, and the second question asked for respondents' academic credentials respondents. The third question asked the respondents to suggest the public health importance and actionability for public health intervention (e.g., education, outreach, testing, vaccination) of 74 targets. Next, respondents were asked to indicate the appropriate level of sampling for each target based on 5 categories: schools (K-12), nursing homes, jails/detention centers, homeless shelters, and citywide. Finally, respondents were asked to identify the top 3 "most important" targets from the list; to provide

any comments about the targets included on the list; and to suggest additional targets that were not included in the list.
